# Immune infiltrating cells in cholangiocarcinoma may become clinical diagnostic markers: based on bioinformatics analysis

**DOI:** 10.1186/s12957-021-02168-8

**Published:** 2021-02-22

**Authors:** Yongwei Zhang, Sihan Chen, Jun Li, Wei Dai, Yeben Qian

**Affiliations:** Department of General Surgery, The First Affiliated Hospital of Anhui Medical University, Hefei, China

**Keywords:** Bioinformatics analysis, Prognosis, Intrahepatic cholangiocarcinoma, Immune cells

## Abstract

**Background:**

Intrahepatic cholangiocarcinoma (ICC) is a malignant tumor originating from the secondary bile duct and its branch epithelium. Among primary liver tumors, the incidence of ICC is second only to hepatocellular carcinoma. Tumor microenvironment can regulate the occurrence and development of tumors. This study is dedicated to finding more markers that can diagnose ICC by finding the differential tumor microenvironment cells between ICC and normal tissues.

**Methods:**

We wanted to study the infiltration of immune cells between the cholangiocarcinoma of the same patient and its paired non-tumor tissues, to explore the difference of immune cells in the tumor microenvironment and adjacent non-tumor tissues in the same organism. So, we searched the relevant data of patients with ICC from the GEO database and found that the GSE45001 data set meets our research needs. CIBERSORT database is used to calculate immune cell composition. Finally, perform visual analysis and data statistics to find out the differentially expressed immune cells.

**Results:**

We found that the expression levels of dendritic cells activated, macrophages M2, and T cells regulatory (Tregs) in ICC were higher than normal tissues, and the expression levels of macrophages M1, monocytes, and T cells follicular helper in ICC were lower than normal tissues.

**Conclusion:**

These 6 types of immune cells are expected to become molecular markers for clinical diagnosis of ICC.

## Background

Intrahepatic cholangiocarcinoma (ICC) is a malignant tumor originating from the secondary bile duct and its branch epithelium [1]. Among primary liver tumors, the incidence of ICC is second only to hepatocellular carcinoma, and the incidence of ICC in China is on the rise. Early diagnosis can give ICC patients better therapeutic effects, so it is very important to find more markers to diagnose ICC [2]. Recent studies have shown that the tumor microenvironment can regulate the occurrence and development of tumors. This study is dedicated to finding more markers that can diagnose ICC by finding the differential tumor microenvironment cells between tumors and normal tissues [3].

In this study, by searching the ICC-related data set in the GEO database, we established relevant selection and exclusion criteria: (1) must be paired intrahepatic cholangiocarcinoma and para-cancer tissue, (2) the quantity must be at least 10 pairs, and (3) these data sets have not previously been used to study immune cell infiltration in ICC [4]. It was found that GSE45001 [5] (https://www.ncbi.nlm.nih.gov/gds/?term=GSE45001) met the research conditions (with 10 tumor samples and 10 normal tissue samples). We put it into the CIBERSORT (https://cibersort.stanford.edu/) [6] database to analyze their immune cell composition. Then, take histograms, principal component maps, heat maps [7], and other visualizations to show the differences in immune cells between tumor tissues and normal tissues. We found that there are indeed some differences in the content of immune cells between ICC tissues and normal tissues, and through co-expression heat maps, violin diagrams, etc., more accurately extract the difference between tumor tissues and normal tissues.

This study is dedicated to finding new markers that can diagnose ICC. The results show that the expression differences between dendritic cells activated, macrophage M1, macrophage M2, monocytes, T cells follicular helper, and T cells regulatory (Tregs) between ICC and normal tissue are statistically significant (*P* < 0.05) are expected to become a new diagnostic ICC marker.

## Methods

### Data collection and download

We searched the relevant data of patients with ICC from the GEO database and found that the GSE45001 data set meets our research needs. It contains data for 10 pairs of tumor tissues and normal tissues.

### Evaluation of the content of immune cells in the sample

CIBERSORT is a database that can calculate the immune cell content of each sample, from which we obtain the information of the immune cell content of the sample we want. We will download 20 samples of the data set and calculate the relevant immune cell content in CIBERSORT.

### Drawing of heat maps related to samples and immune cells

Use the R language pheatmap package to analyze and draw a heat map of the relationship between immune cells and sample expression.

### Drawing and analysis of the correlation heat map of immune cells

The corrplot package of the R language was used to analyze the correlation of the immune cells expressed in the samples in this study and draw a correlation heat map.

### The violin diagram of the expression of immune cells in adjacent tissues and ICC tissues

Use the vioplot package of R language to analyze the sample’s immune cells and draw a violin chart (Wilcoxon test was used for statistical analysis, *P* value has been adjusted). Therefore, yellow represents the tumor tissue group, and green represents the normal tissue group.

### The paired difference graphs explain the expression of immune cells in the sample

Use R language related codes to draw paired difference maps. The two shapes connected by black lines are two types of tissues of the same patient.

### Survival analysis and Kaplan-Meier curve drawing

Analyze the direct relationship between immune cells and survival prognosis in Tumor Immune Estimation Resource (http://timer.cistrome.org/) (TIMER 2.0) [8, 9] database and draw K-M curve.

### Sample principal component analysis chart

Use R language code, the main component analysis of the sample. Use two circles to distinguish between the tumor group and the normal group, and one dot represents one sample.

### Statistics description

The statistical method of the violin diagram and the paired difference graphs are: use the Wilcoxon signed-rank test to calculate the difference in immune cell content between tumor tissue and normal tissue. Kaplan-Meier curves were used to evaluate the relationship between the survival data of each variable. *P* < 0.05(*) is considered statistically different. *P* < 0.01(**) is considered to have a significant statistical difference.

## Result

### Download and analysis of data

We searched the GEO database for samples that meet the research requirements and found that GSE45001 meets our needs. It contains 10 ICC samples and 10 normal samples (Table 1).

**Table 1:** Data from GSE45001; 20 samples from 10 paired CCA patients

GSM1095633	Normal stroma of CCA	GSM1095634	Tumoral stroma of CCA
GSM1095635	Normal stroma of CCA	GSM1095636	Tumoral stroma of CCA
GSM1095637	Normal stroma of CCA	GSM1095638	Tumoral stroma of CCA
GSM1095639	Normal stroma of CCA	GSM1095640	Tumoral stroma of CCA
GSM1095641	Normal stroma of CCA	GSM1095642	Tumoral stroma of CCA
GSM1095643	Normal stroma of CCA	GSM1095644	Tumoral stroma of CCA
GSM1095645	Normal stroma of CCA	GSM1095646	Tumoral stroma of CCA
GSM1095647	Normal stroma of CCA	GSM1095648	Tumoral stroma of CCA
GSM1095649	Normal stroma of CCA	GSM1095650	Tumoral stroma of CCA
GSM1095651	Normal stroma of CCA	GSM1095652	Tumoral stroma of CCA

### Analysis of the content of immune cells in the sample

**Fig. 1:**
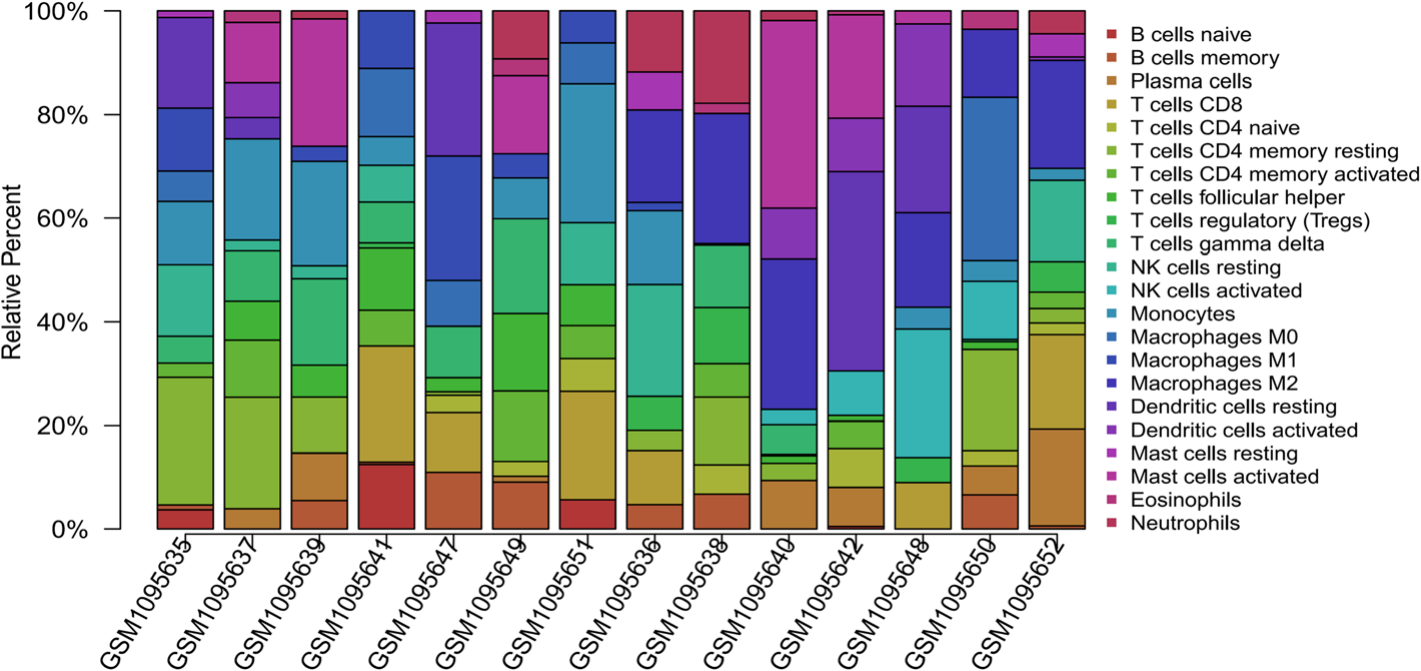
The 7 pairs of data sets were analyzed for immune components and plotted as a histogram. From left to right, the first 7 GSM samples are normal tissues, and the last 7 GSM samples are tumor tissues

**Fig. 2:**
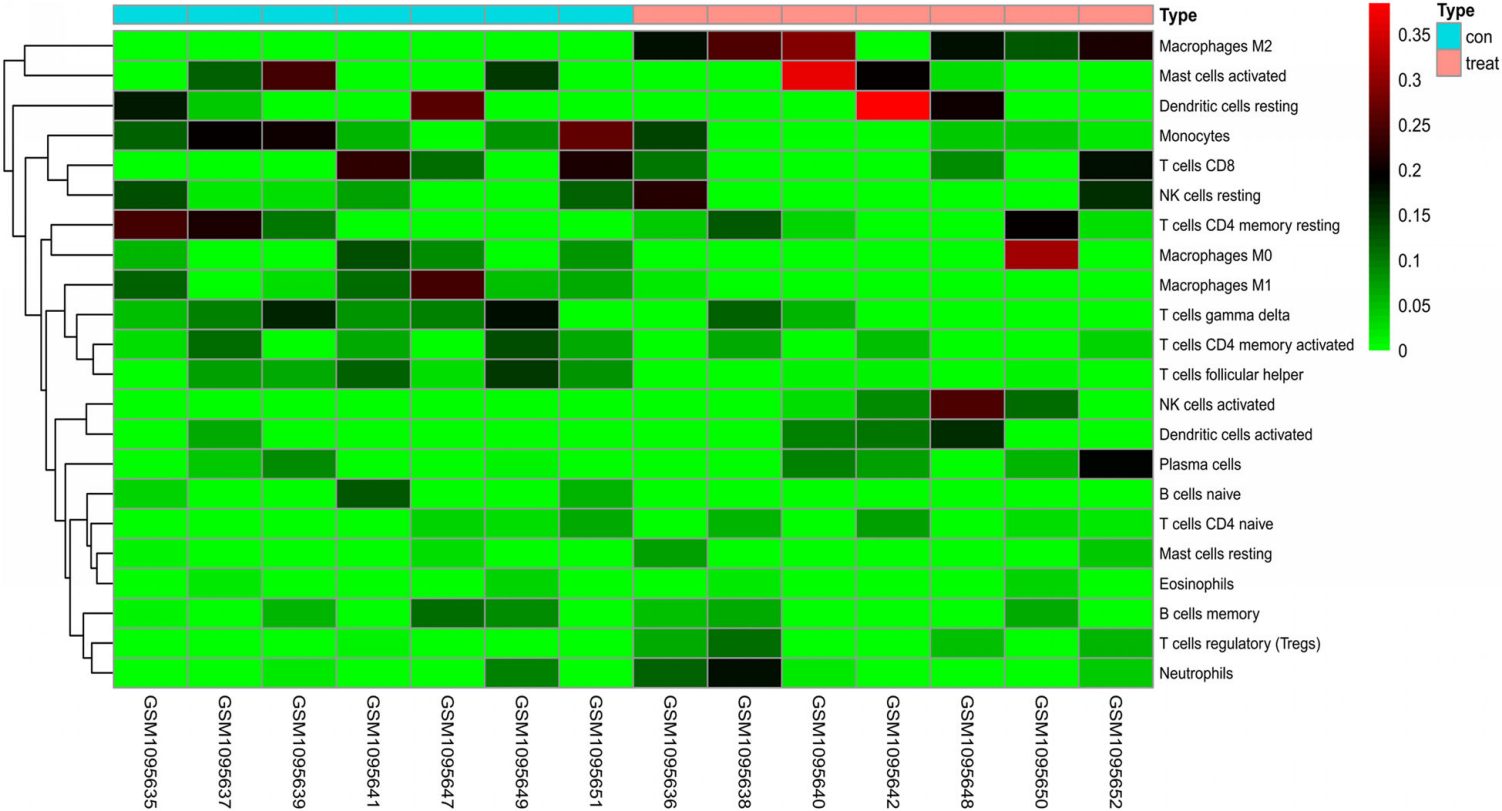
A heatmap of the 22 immune cell proportions based on GEO data. Red indicates upregulation and green indicates downregulation. Explanation of “type”: red represents tumor tissue and blue represents normal tissue

After quality inspection, we found that 7 pairs of samples were available, and we downloaded them and included them in the study. The 7 normal samples and 7 ICC samples obtained by the correction were put into the CIBERSORT database to calculate the immune cell content, and the obtained results were plotted as a histogram. Then, we further draw the results into a principal component diagram, from which we can find that the tumor tissue group and the normal tissue group have significantly different immune cell content (Fig. 1). Finally, we draw a heat map and visually analyze the expression of immune cells in the sample (Fig. 2). The results show that the expression of some immune cells shows differences between tumor tissues and normal tissues.

### Co-expression analysis between immune cells

**Fig. 3:**
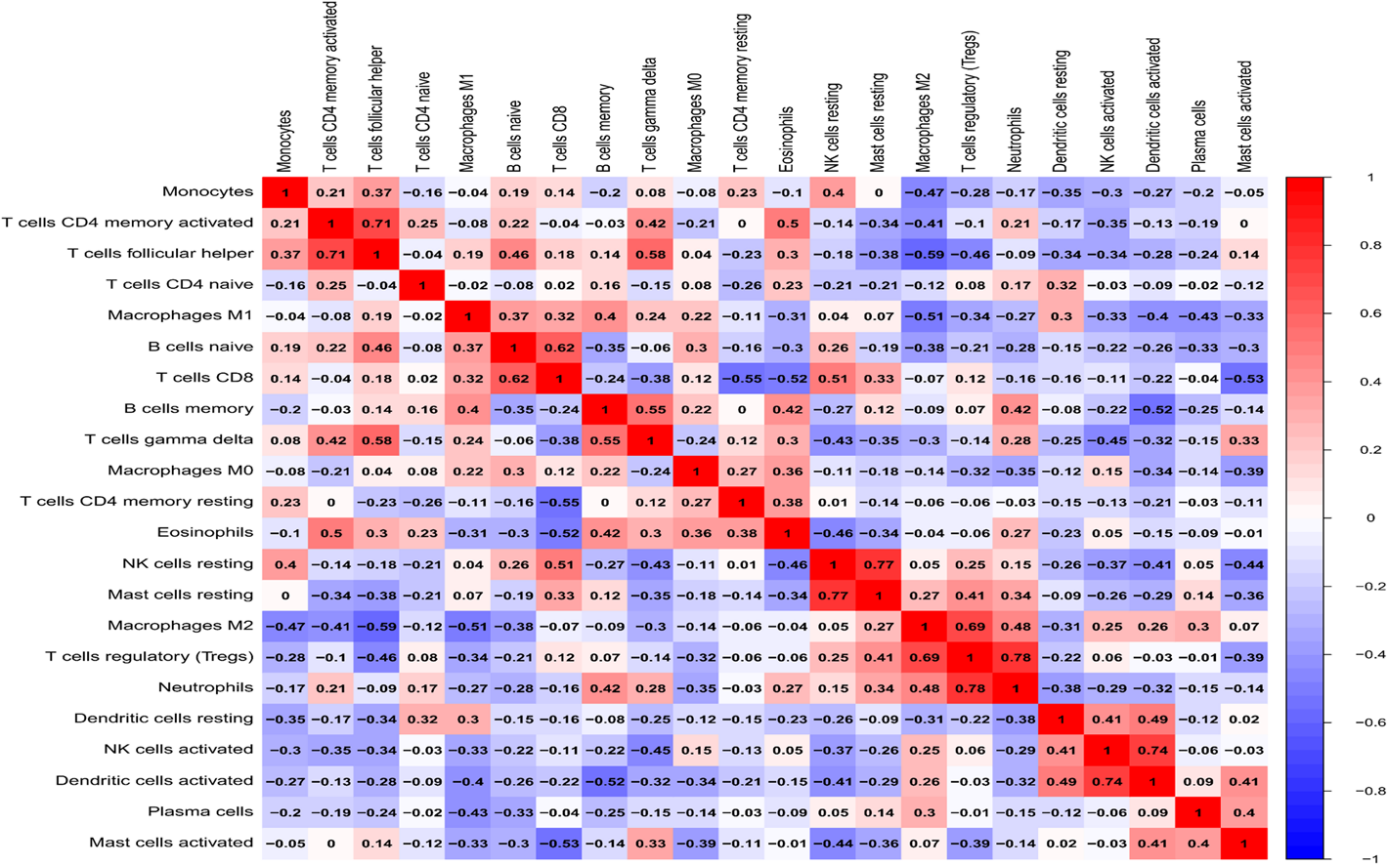
Correlation matrix of 22 immune cell proportions calculated by CIBERSORT software. The *X*-axis and *Y*-axis indicate the types of immune cells, and the *Z*-axis indicates the level of correlation between the two immune cells (red represents the stronger the correlation, and blue represents the lower the correlation)

Co-expression analysis of the expression levels of immune cells obtained in this study showed that the correlation between T cells regulatory (Tregs) [10–13] and neutrophils [14–17] was the strongest, with a coefficient of 0.78, and the two cells with the weakest correlation were macrophages [18, 19] and T cells follicular helper [20, 21]; the correlation coefficient is − 0.59 (Fig. 3).

### Immune cells with significantly different expressions in ICC and normal tissues are screened

**Fig. 4:**
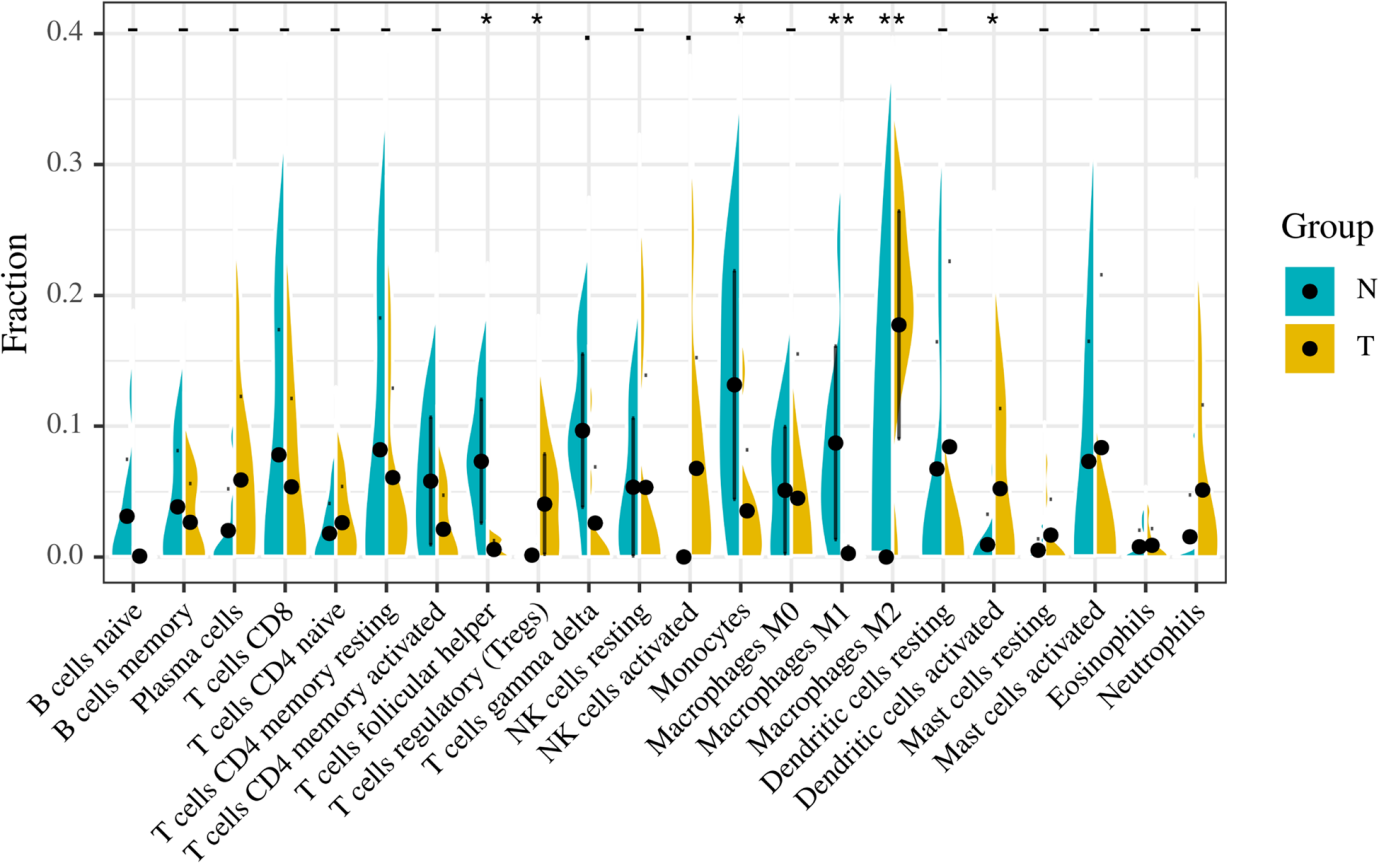
Comparison of immune cell content between normal tissue and ICC tissue. *P* < 0.05(*) is considered statistically different. *P* < 0.01(**) is considered to have a significant statistical difference

**Fig. 5:**
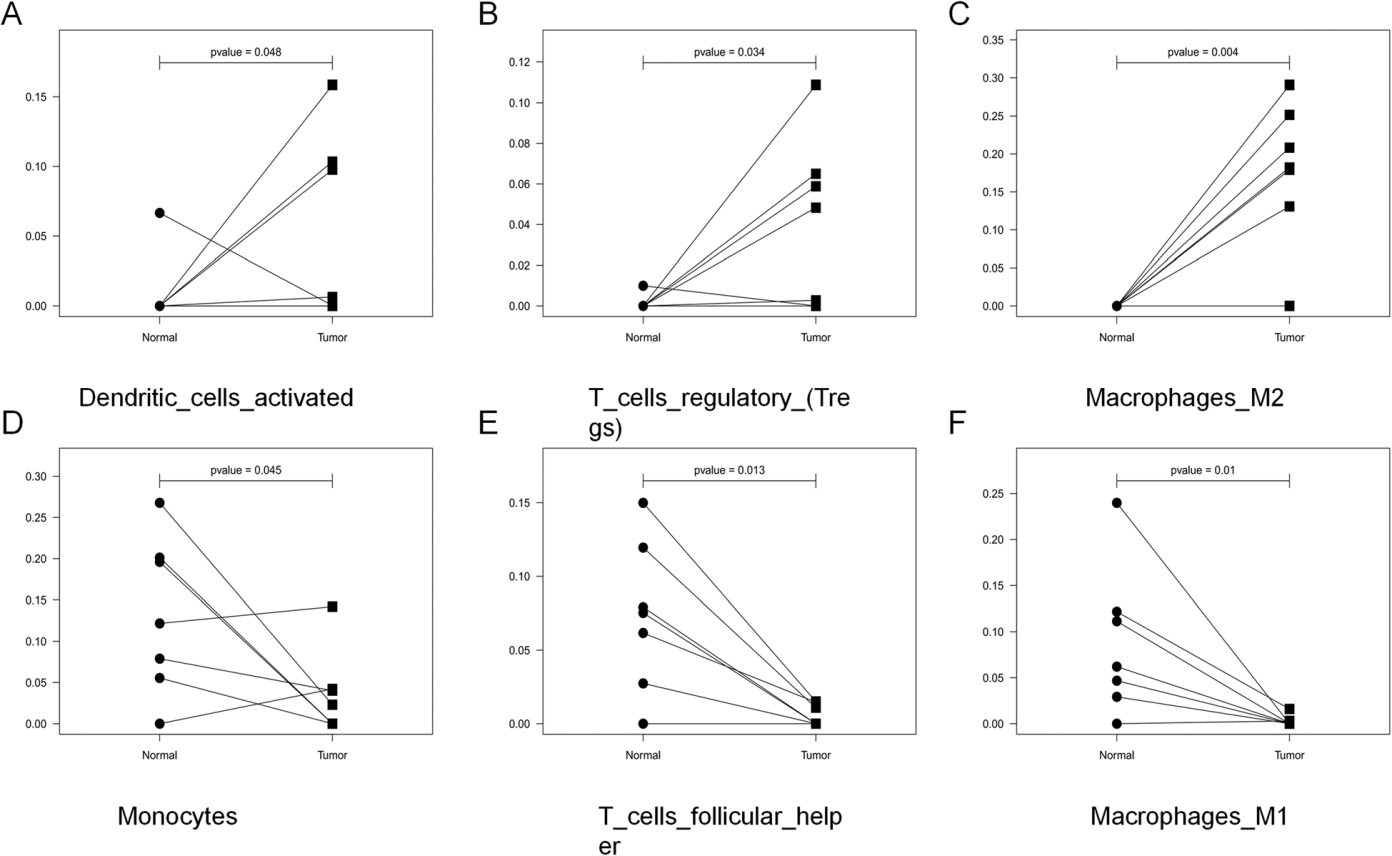
Dendritic cells, T cells regulatory, and macrophages M2 augment, while monocytes, T cells follicular and macrophages M1 decrease from normal to tumor microenvironments. *P* < 0.05 is considered statistically different. *P* < 0.01 is considered to have a significant statistical difference

By drawing a violin chart (Fig. 4), We found that the expression levels of dendritic cells activated (Fig. 5a), macrophages M2 (Fig. 5b), and T cells regulatory (Tregs) (Fig. 5c) in ICC were higher than normal tissues and the expression levels of monocytes (Fig. 5d), T cells follicular helper (Fig. 5e), and macrophages M1 (Fig. 5f) in ICC were lower than normal tissues.

### Principal component analysis (PCA)

**Fig. 6:**
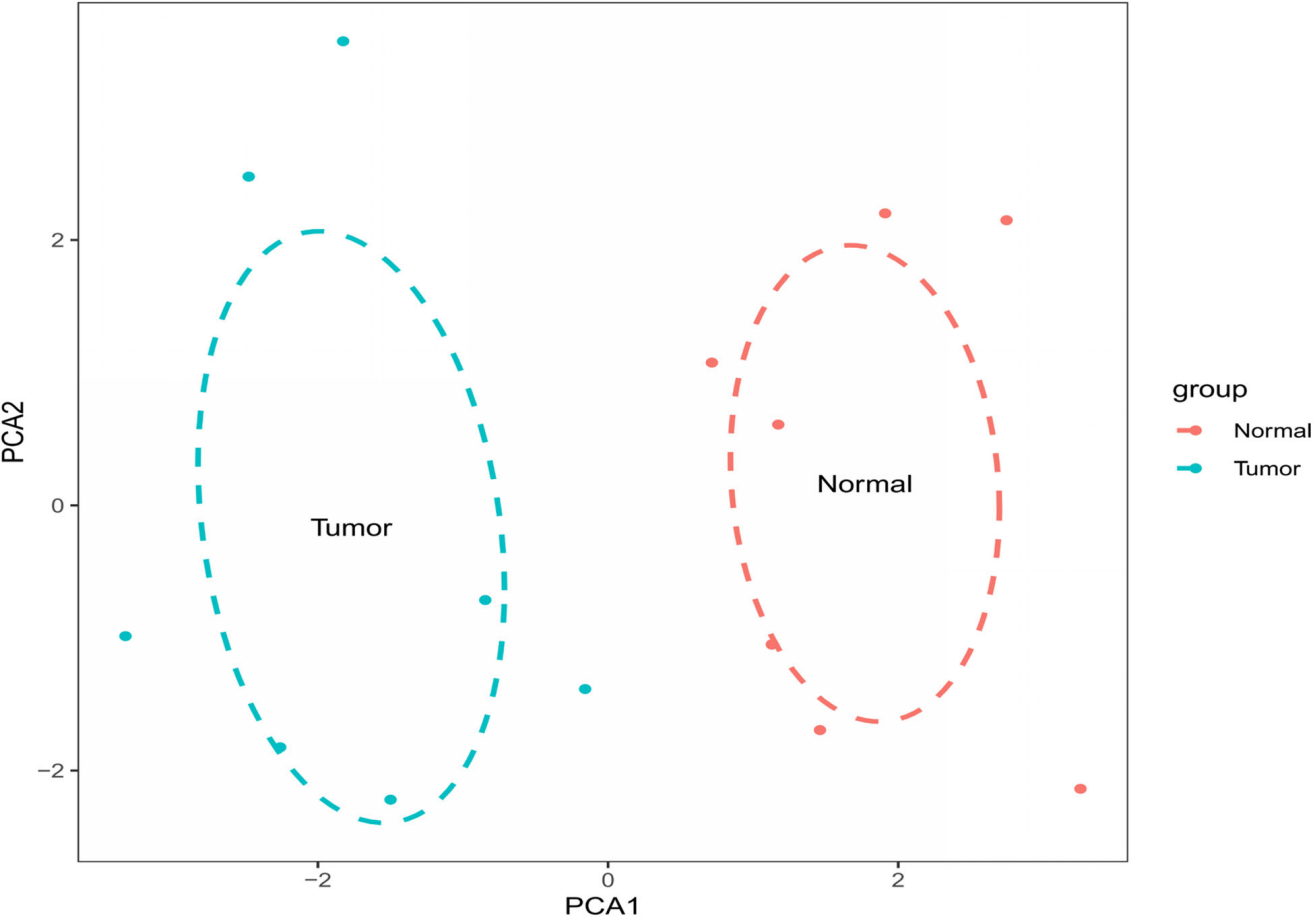
PCA (principal component analysis). The two ellipses did not intersect, indicating that 22 kinds of immune cells could well distinguish tumor group from normal group. The red color represents the normal group, and the blue color represents the tumor group

After obtaining the matrix of immune cells, we wondered whether these immune cells could distinguish between the normal group and the tumor group. Then we did principal component analysis. The dimensionality was reduced to PCA1 and PCA2 by PCA, and the *X*-axis was labeled as PCA1 and *Y*-axis as PCA2. Then an ellipse was simulated for the normal group and the tumor group, respectively. If the two ellipses did not cross, it suggested that the 22 immune cells could distinguish the normal group from the tumor group well. We used the “GGplot2” package for analysis, and the results showed that the two ellipses did not cross (Fig. 6), indicating that the 22 kinds of immune cells in this study could well distinguish the tumor group from the normal group.

### Immune cells related to ICC survival prognosis

**Fig. 7:**
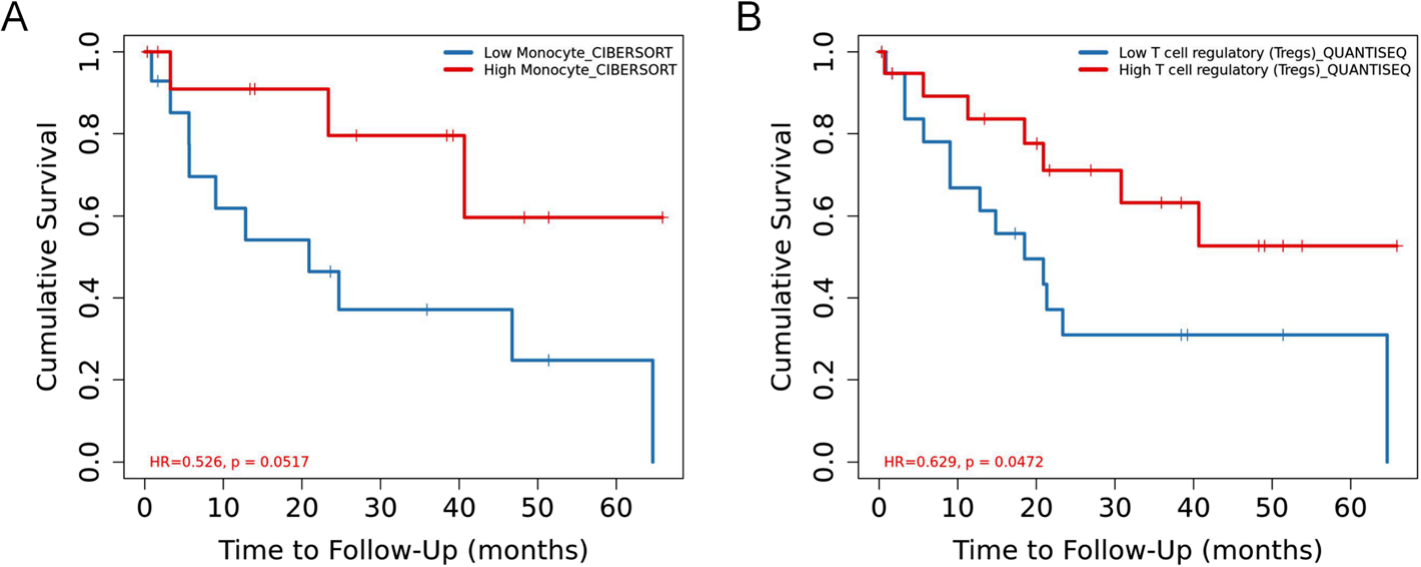
Lower infiltration of monocyte (**a**) and T cell regulatory (**b**) fraction was associated with poor overall survival in patients with ICC. *P* < 0.05 is considered statistically different

We further explore the relationship between each immune cell in Fig. 7 and ICC survival in the Timer2.0 database. It was found that the survival rate of the group with high monocyte cell content was significantly better than that of the group with low monocyte cell content.

The survival rate of the group with high T cell regulatory content was better than that of the group with low regulatory content. However, the data of other cells is not sufficient at present, and further research and supplement are needed.

## Discussion

Intrahepatic cholangiocarcinoma (ICC) is a type of primary malignant tumor of the liver that originates from the bile duct and its branches. Because the clinical symptoms of ICC are not obvious early, it often causes patients to lose the opportunity for timely surgery. Therefore, it is extremely critical to find a better method for early diagnosis of ICC. The tumor microenvironment is closely related to the occurrence and development of tumors. Many studies have shown that the tumor microenvironment plays a role in promoting tumorigenesis and development, such as promoting angiogenesis, producing growth factors that promote tumors, and causing hypoxic environments and inflammatory reactions. However, ICC-related research has not explored the relationship between the tumor microenvironment and ICC. With the development of biochip technology, more and more bioinformatics databases and bioinformatics tools are available for our research and use. Therefore, we can analyze the cells that constitute the immune microenvironment of ICC through bioinformatics technology to find tumor markers that can better diagnose ICC.

In this study, we retrieved the ICC data set from the GEO database and found that GSE45001 met our research needs. Then we downloaded and grouped them reasonably, 10 samples were classified into tumor group, and 10 samples were classified into normal tissue group. Put these 20 samples into the CIBERSORT database to analyze the content of immune cells and draw the principal component map. The results show that the composition of immune cells between tumor tissues and normal tissues is not the same. Through the heat map we draw, we can find that some immune cells are expressed in tumors higher than normal tissues, but some immune cells are lower than normal tissues. From the co-expression heat maps we draw, we can better discover which immune cells are more or less relevant in this study, and drawing a violin chart can more intuitively find the expression of each immune cell in different tissue. Finally, we found that the expression of dendritic cells activated, and macrophages M2, T cells regulatory (Tregs) in tumor tissue was higher than that in normal tissue, while the expression of macrophages M1, monocytes, and T cells follicular helper in tumor tissue was lower than that in normal tissue. This difference provides us with more basis for clinical diagnosis of ICC.

Dendritic cells have long been considered to be anti-tumor immune cells, but studies have found that dendritic cells can promote tumor suppression of immunity. Some people have achieved the idea of inhibiting tumor growth by suppressing the number of dendritic cells and believe that dendritic cell-related pathways may become new therapeutic targets [22]. Macrophages M1 cells can accelerate the apoptosis process of tumor cells, in line with our research results, its expression in normal tissues is higher, and it has a certain effect on inhibiting tumor cells. However, macrophages M2 and macrophages M1 have opposite functions. They can delay the apoptosis of tumor cells and promote the progression of tumors [23]. Monocytes can not only promote tumor growth by increasing angiogenesis, but also inhibit tumor growth by identifying and clearing tumor escape signal pathways. In this study, although the content of monocytes was higher in normal tissues than in tumor tissues in general, there were obviously some of the opposite. According to our statistical analysis, monocytes inhibit tumor growth in ICC [24]. By querying in the TIMER 2.0 database, two immune cells (monocytes, T cells regulatory) were found to be related to the survival prognosis of ICC patients, and there was statistical significance. And these two cells also fit the logic of this study that both of these cells are protective factors for ICC.

## Conclusion

In this study, we analyzed the components of the immune microenvironment of ICC and found that 6 types of immune cells are differentially expressed between ICC and normal tissues. These 6 types of immune cells are expected to become molecular markers for clinical diagnosis of ICC.

## Data Availability

The data used to support the findings of this study are included within the article.

## Abbreviations

ICC: Intrahepatic cholangiocarcinoma
Tregs: T cells regulatory
TIMER: Tumor Immune Estimation Resource
K-M curve: Kaplan-Meier curve
PCA: Principal component analysis
